# Extracellular vesicles in oral squamous cell carcinoma: current progress and future prospect

**DOI:** 10.3389/fbioe.2023.1149662

**Published:** 2023-05-26

**Authors:** Yanqi Zhang, Jianing Liu, Shiyu Liu, Lu Yu, Siying Liu, Meng Li, Fang Jin

**Affiliations:** ^1^ Department of Orthodontics, School of Stomatology, The Fourth Military Medical University, Xi’an, China; ^2^ State Key Laboratory of Military Stomatology, National Clinical Research Center for Oral Diseases, Shaanxi International Joint Research Center for Oral Diseases, Center for Tissue Engineering, School of Stomatology, The Fourth Military Medical University, Xi’an, China; ^3^ Department of Periodontology, Shandong Key Laboratory of Oral Tissue Regeneration, Shandong Engineering Laboratory for Dental Materials and Oral Tissue Regeneration, Shandong Provincial Clinical Research Center for Oral Diseases, School and Hospital of Stomatology, Cheeloo College of Medicine, Shandong University, Jinan, Shandong, China; ^4^ Department of Prosthodontics, School of Stomatology, The Fourth Military Medical University, Xi’an, China

**Keywords:** oral squamous cell carcinoma, extracellular vesicles, diagnosis, tumorigenesis, treatment

## Abstract

Oral squamous cell carcinoma (OSCC) is the most aggressive oral and maxillofacial malignancy with a high incidence and low survival rate. OSCC is mainly diagnosed by tissue biopsy, which is a highly traumatic procedure with poor timeliness. Although there are various options for treating OSCC, most of them are invasive and have unpredictable therapeutic outcomes. Generally, early diagnosis and noninvasive treatment cannot be always satisfied simultaneously in OSCC. Extracellular vesicles (EVs) are involved in intercellular communication. EVs facilitate disease progression and reflect the location and status of the lesions. Therefore, EVs are relatively less invasive diagnostic tools for OSCC. Furthermore, the mechanisms by which EVs are involved in tumorigenesis and tumor treatment have been well studied. This article dissects the involvement of EVs in the diagnosis, development, and treatment of OSCC, providing new insight into the treatment of OSCC by EVs. Different mechanisms, such as inhibiting EV internalization by OSCC cells and constructing engineered vesicles, with potential applications for treating OSCC will be discussed in this review article.

## 1 Introduction

Oral squamous cell carcinoma (OSCC) is a malignant tumor mainly originating from squamous epithelial cells. In 2018, there were 354,864 new cases and 177,384 deaths of OSCC (Bray et al., 2018). OSCC is usually diagnosed in advanced stages, which leads to a poor prognosis. Researchers attempted to find new methods for early diagnosis of OSCC. Currently, the main treatment of OSCC is still surgery combined with chemotherapy, and the main chemotherapy regimen includes 5-fluorouracil (5-FU) and cisplatin (Longley et al., 2003). However, 5-year survival remained low, mainly due to chemoresistance (Epstein et al., 2012; Chi et al., 2015). Therefore, it is crucial to improve the diagnostic approach and treatment method in OSCC.

**FIGURE 1:**
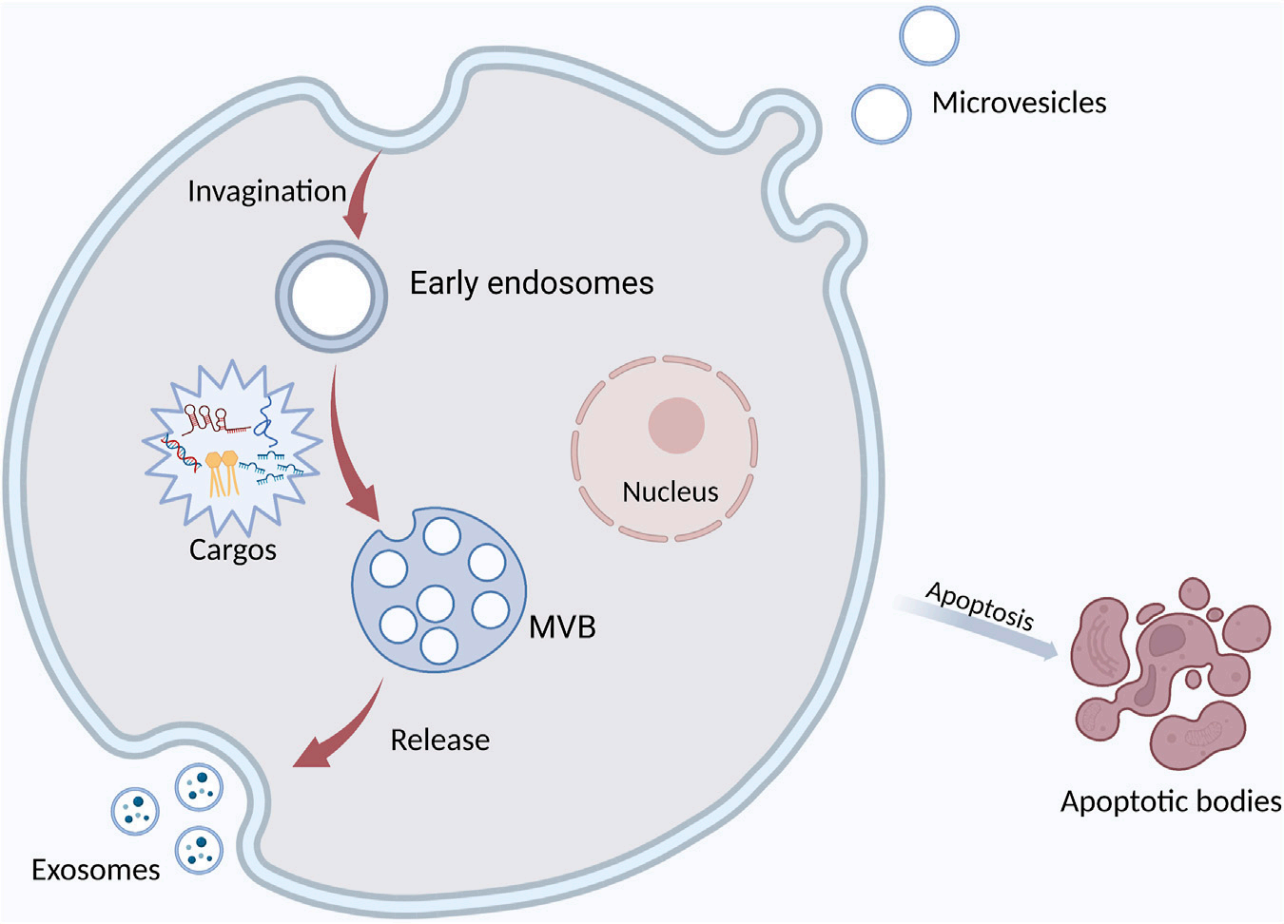
The formation and secretion of EVs. Extracellular vesicles are mainly classified into three main types: exosomes, microvesicles, and apoptotic bodies. Exosomes are formed by the fusion of multivesicular bodies (MVBs) with the plasma membrane, MVs form directly from the plasma membrane to the outward bud, and apoptotic bodies are released by the cells after apoptosis. It contains DNA, miRNA, protein, and other cargos.

EVs have been a main focus of research in recent years. They are membrane-derived vesicles secreted by different cells and widely found in body fluids. According to their biogenesis and releasing mode, EVs can be divided into microvesicles, exosomes, and apoptotic bodies. They carry DNA, RNA, protein, miRNA, and other substances (Doyle and Wang, 2019) (Figure 1). EVs widely exist in tumor microenvironment (TME) and participate in cellular communication. EVs have the potential to be used for minimally invasive diagnosis because they are involved in tumorigenesis and they contain is a biologic sample from tumor cells. EVs exist in various body fluids and are easy to obtain. In addition, EVs regulate cell function by delivering their vesicular substances, thus participating in the development of OSCC (van Niel et al., 2022). More and more attention has been recently paid to the role of EVs in tumorigenesis (Brinton et al., 2015; Bebelman et al., 2018; Urabe et al., 2020). EVs are involved in many important processes in OSCC (Kaiser, 2016), such as angiogenesis and epithelial-mesenchymal transformation (EMT). Tumor-associated macrophages (TAM) are the main cell in TME and promote tumor development. EVs can regulate the phenotypic transition of macrophages. EVs are internalized by macrophages, and their signaling molecules regulate the polarization of macrophages.

In this review, we have discussed how EVs are involved in the diagnosis and development of OSCC. By understanding the role of EVs in the development of OSCC, it would be possible to design more effective therapeutic regimens. Moreover, by modifying the surface characteristics and contents of EVs, their targeting ability and treatment efficiency can improve (Herrmann et al., 2021). Therefore, producing manipulated EVs is a promising method for treating OSCC. So far, few review articles comprehensively analyzed the efficacy of EVs in the diagnosis, tumorigenesis, and treatment of OSCC, and most of them only analyzed some of these topics. Therefore, we summarized the role of EVs in the development, diagnosis, and treatment of OSCC. In addition, whether EVs can improve the survival rate of OSCC patients is also discussed.

## 2 The applications of EVs in the diagnosis and prognosis of OSCC

EVs contain several types of biological molecules, including proteins, lipids, DNA, and RNA, which play important roles in the development and progression of OSCC. Particularly, they are involved in several biological processes such as proliferation, apoptosis, DNA repair, metabolism, angiogenesis, and immune response (Becker et al., 2016; Ciardiello et al., 2016; Almeria et al., 2019; Kalluri and LeBleu, 2020). Some constituents of EVs are upregulated and others are downregulated to contribute to tumor cell proliferation and invasion. Non-coding RNAs, including miRNA, lncRNA, and circ RNA, along with proteins, have been increasingly investigated as diagnostic and prognostic markers for OSCC. Apart from tissue specimens, diagnostic EVs are most often collected from body fluids, such as saliva and serum, or cell culture supernatants. Body fluid-derived EVs can be easily used for minimally invasive early diagnosis and prognosis (Li et al., 2021).

### 2.1 RNAs in EVs as diagnostic markers for OSCC

Exosomal RNAs are heavily involved in the interaction between tumor cells and TME (Table 1). Carcinoma-derived exosomal RNAs can promote TME remodeling, and exosomal RNAs originating from TME can also affect tumor cell behavior. By regulating tumor cell-TME crosstalk, exosomal RNAs can facilitate tumorigenesis, tumor growth, and metastasis (Fang et al., 2020; Tan et al., 2020; Forder et al., 2021; Lu et al., 2021; Villegas-Pineda et al., 2021; Zhao et al., 2022). Therefore, RNAs in EVs have increasingly received attention for OSCC diagnosis.

**TABLE 1:** Exosomal RNA biomarkers related to diagnosis and prognosis of OSCC.

Biomolecules	Molecules	Origin	Findings	Ref(s)
OncomiRs				
	miR-24-3p	OSCC patients	Elevate in salivary exosomes of OSCC patients (AUC = 0.738)	He et al. (2020a)
	miR-23a-3p	SCC-9 and CAL-27	OSCC cells derived exosomes enriched than OSCC cells	Cai and Qiao (2019)
	miR-155, miR-21	serum samples of OSCC patients	upregulated in exosomes derived from primary tumor cells from OSCC patients than paired normal cells	Chen and Huang (2021)
	miR-342-3p, miR-1246	HOC313-LM	express high in exosomes and promote its motility	Sakha et al. (2016)
	miR-30a	OSCC patients	a positive relation between the expression in exosomes and tissues of OSCC patients (AUC = 0.812)	He et al. (2021b)
	miR-302b-3p, miR-517b-3p	Saliva of OSCC patients	only discovered in EVs from OSCC patients saliva but healthy controls	Gai et al. (2018)
	miR-512-3p, miR-412-3p	OSCC patients	increase in comparison with the paired	Gai et al. (2018)
	miR-210	plasma of OSCC patients	express higher than normal samples with AUC of 0.9513	Bigagli et al. (2021)
	miR-365	SCC25, CAL27	exist in OSCC cell lines and extracellular vesicles	Coon et al. (2020)
	miR-21	SCC-9, CAL-27	drive recipient cells toward EMT, advantageous for tumor migration and invasion	Li and Zhu (2016)
TSmiRs				
	miR-126	serum exosomes of OSCC patients	suppress angiogenesis, lymphangiogenesis and tumorigenesis by targeting EGFL7	Chen and Huang (2021)
	miR-101-3p	CAL27, SCC9, TCA8113	suppress OSCC progression	Xie et al. (2019a)
Others				
	circ_0000199	OSCC cells from human sample	elevate in OSCC patients, associated with TNM stage, tumor size, lymphatic metastasis	Luo et al. (2020)
	lncRNA HOTTIP	FaDu, CNE-2Z, Hep-2	inhibit HNSCC proliferation through M1 polarization	Jiang et al. (2022)
	lncRNA ADAMTS9-AS	CAL-27, SCC-9	interect with miRs to regulate EMT and AKT signalling pathway and suppresses tumor growth, invasion and metastasis	Zhou et al. (2021)
	lncRNA LBX1-AS1	THP-1, SCC-4, CAL-27	inhibit tumor progression by regulate downstream FOXO expression	Ai et al. (2021)
	lncRNA FLJ22447	HSC-3	ingested by stromal CAFs to upregulate Lnc-CAF in turn, and co-express with IL-3 to promote OSCC growth	Ding et al. (2018)

#### 2.1.1 Correlation between EV-derived miRNAs and OSCC

miRNAs, also known as small non-coding RNAs, have 19–24 nucleotides and post-transcriptionally regulate gene expression in several physiological and pathological processes (Lu et al., 2021). Circulatory miRNAs exist in three forms: free circulating miRNAs, exosomal miRNAs, and conjugated with argonaute proteins (Ago2) (Sahu and Routray, 2021). Free miRNAs in saliva, serum, and plasma can accelerate the less-invasiveness diagnosis of OSCC and improve patients’ compliance compared with conventional methods like tissue biopsy and mucosal scraping. Therefore, they received significant attention as a diagnostic method for oral cancer in recent years (He L. et al., 2020). Exosomes contain complex payloads encapsulated by a lipid bilayer, which improves the stability of miRNAs and allows them to resist nucleases (Sahu and Routray, 2021). Recent studies have elucidated that exosomes are deeply involved in the regulation of intercellular communication (Kalluri and LeBleu, 2020). Exosomal miRNAs modulate various disease progression by delivering signaling molecules and miRNAs. Oncogenic miRNAs (oncomiRs) and tumor-suppressing miRNAs (TS-miRNAs) are two subtypes of miRNAs that can be distinguished based on their regulatory roles in oncogenesis (Chen and Huang, 2021).

Exosomal miRNAs with differential expression in OSCC cells and normal cells have been the focus of research in recent years. Salivary exosomes from OSCC patients and exosomes derived from OSCC tissues had significantly higher levels of miR-24-3p compared with normal controls. The ROC curve revealed that the area under the curve (AUC) for salivary exosomes was 0.738 (He L. et al., 2020). Therefore, miR-24-3p in salivary exosomes can potentially help the early diagnosis of OSCC (He L. et al., 2020). As a potential diagnostic biomarker, miR-30a was significantly overexpressed in exosomes and OSCC tissues, with an AUC value of 0.812. A high level of miR-30a also predicted shorter recurrence-free survival (He T. et al., 2021). Similarly, miR-210 had significantly higher levels in plasma-derived EVs from OSCC patients than in plasma-derived EVs from control samples (AUC = 0.951) (Bigagli et al., 2021). miR-365 was expressed in OSCC cell lines and transferred into EVs produced by OSCC cell lines (Coon et al., 2020). Similar to miR-365, miR-23a-3p was significantly upregulated in OSCC cell lines (SCC-9 and CAL-27). Intriguingly, exosomes derived from OSCC cell lines contained higher levels of miR-23a-3p compared with OSCC cells. miR-23a-3p-rich tumor-derived exosomes (TEX) can induce macrophage polarization toward the M2 subtype, associated with tumor growth and invasion (Cai and Qiao, 2019). A miRNA array confirmed that compared with conjugated normal cells, oncogenic miR-155 and miR-21 were upregulated by two to five folds in exosomes derived from primary OSCC cells. They promote cell proliferation and invasion by downregulating PTEN and Bcl-6 tumor suppressor genes in OSCC cells (Chen and Huang, 2021). miR-21-5p is the most abundant microRNA (miR) in EVs released from OSCC cells. miR-21-5p promotes tumor cell stemness and migration (Chen et al., 2019). Two key oncomiRs, miR-342-3p and miR-1246, are highly expressed in OSCC-derived exosomes. They were transferred to HOC313-P cell and enhanced its motility by exosomes derived from HOC313-LM cell, a type of human OSCC cell line with high metastatic capacity (Sakha et al., 2016). miR-302b-3p and miR-517b-3p were only detected in salivary EVs from OSCC patients. miR-512-3p and miR-412-3p had higher expression levels in OSCC patients (Gai et al., 2018). In addition, the oxygen concentration in TME can affect the contents of OSCC-derived exosomes. OSCC-derived exosomes can enhance the metastasis and invasiveness of OSCC cells under hypoxic conditions (Li and Zhu, 2016). miRNA sequencing explains how exosomes from OSCCs in normoxic and hypoxic environments differently express miRNAs. Of 108 differentially expressed miRNAs in OSCCs, miR-21 exhibited the greatest difference. It was upregulated in OSCCs and induced EMT, which is needed for tumor migration and invasion. Exosomal miR-21 level may be used as a diagnostic marker in OSCC (Li and Zhu, 2016). Various onco-miRNAs are upregulated in OSCC-derived EVs, which can serve as diagnostic markers.

Although exosomal miRNAs have several advantages as OSCC biomarkers, their application is still controversial because the sensitivity of isolation methods alters the composition of exosomes. Furthermore, smoking, alcohol abuse, and HPV infection can upregulate exosomal miRNAs in the serum samples of healthy participants. Moreover, exosomal miRNAs can be upregulated in other malignant tumors like breast cancer, bladder cancer, hepatocellular carcinoma, and Hodgkin’s lymphoma (He L. et al., 2020).

On the other hand, exosomal miR-126, a TS-miRNA, is downregulated in OSCC. miR-126 downregulates epidermal growth factor-like domain multiple 7 (EGFL7), a tumor-suppressor in OSCC, thereby regulating vascular endothelial growth factor (VEGF), Notch, and Wnt signaling pathways. (Chen and Huang, 2021). Previously, it was found that miR-101-3p has significantly higher expression in normal tissues compared with adjacent malignant tissues. Similarly, it was shown that miR-101-3p is downregulated in several OSCC cell lines such as CAL27, SCC9, and TCA8113 (Xie et al., 2019a, 101-3). According to *in-vitro* and *in-vivo* experiments, exosomes transfer miR-101-3p from hBMSCs to OSCC cells to prevent OSCC progression (Xie et al., 2019a). Although TS-miRNAs have not yet been investigated comprehensively, they are promising for both diagnosis and treatment of OSCC.

#### 2.1.2 The value of other non-coding exosomal RNAs in the diagnosis of OSCC

It is worth noting that other exosomal non-coding RNAs always function through interaction with miRNAs. Bioinformatics analysis revealed that exosomal circ_0000199, a circRNA originating from the end of an RNA molecule, sponges particular miRNAs, such as miR-145-5p and miR-29b-3p, to upregulate oncoproteins in OSCC cells. Researchers found that exosomal circ_0000199 expression is associated with TNM stage, tumor size, and lymphatic metastasis and that circ_0000199 overexpression shortens the survival of OSCC patients (HR 3.57; 95% CI 2.48-6.24, *p* = 0.0035) (Luo et al., 2020). M1 macrophage-derived exosomal lncRNA HOTTIP can decrease proliferation, induce apoptosis, and enhance M1 phenotype differentiation in head and neck squamous cell carcinomas (HNSCC) (Jiang et al., 2022). Exosomal lncRNA LBX1-AS1 is released by RBPJ-OE macrophages. It competes with miR-182-5p to prevent the growth of tumor cells by regulating Forkhead Box O (FOXO) expression (Ai et al., 2021, 1). lncRNA Lnc-CAF is remarkably upregulated in HSC3 cell-derived exosomes. It can be absorbed by cancer-associated fibroblasts (CAFs) to upregulate Lnc-CAF. This forms a positive feedback loop and causes Lnc-CAF co-expression with IL-3, which promotes OSCC growth (Ding et al., 2018). Similar to miRNAs, other non-coding RNAs can help in the early detection of OSCC. However, there are only a few studies investigating the role of other types of exosomal non-coding RNAs in OSCC. circ IGHG is highly expressed in OSCC and induces EMT, suggesting a promising potential for theranostics, but still, further studies are needed (Liu et al., 2021).

### 2.2 Cargo proteins and surface markers of EVs are indicators of OSCC

Previous articles have reviewed several proteins used as hallmarks of OSCC prognosis (Sasahira and Kirita, 2018); nevertheless, exosomal proteins are extensively diverse and our knowledge about their functions is rapidly growing. Intriguingly, it was unfolded that PTEN, a crucial tumor suppressor protein for angiogenesis, is downregulated by miR-130b-3p in OSCC cells; however, PTEN mRNA level did not significantly change compared with adjacent healthy tissues (Yan et al., 2021). It has been suggested that PTEN and PDCD4 both downregulate STAT3 (Chen et al., 2019). STAT3 silencing reduced miR-21-5p level (Chen et al., 2019), which has been introduced as an oncomiR in previous sections. SWATH-MS analysis between healthy, OSCC_NLNM, and OSCC_LNM groups, showed that small proline-rich protein 3 (SPRR3) is significantly upregulated in the salivary small extracellular vesicles (S/SEVs) of OSCC-free participants (Fontana et al., 2021). Recently findings indicated how low expression of SPRR3 is involved in OSCC progression (Yu et al., 2020). In addition, the SWATH-MS analysis uncovered that some proteins (like lipocalin-2, LCN2, and S100) related to anti-microbial defense and inflammatory response are enriched in the S/SEVs of OSCC patients with lymph node metastasis (Fontana et al., 2021). Other studies also unveiled that they can serve as diagnostic and prognostic biomarkers of OSCC (Raffat et al., 2018; Rahimi et al., 2020). Laminin-332 was upregulated in EVs produced by OSCC cell lines (LN1-1) and in plasma EVs derived from OSCC patients with lymph node metastasis. After being absorbed by lymphatic endothelial cells (LECs), Laminin-332 promotes LEC migration and tube formation (Wang et al., 2019). ApoA1, CXCL7, PF4V1, and F13A1 have potential application as novel circulating biomarkers of OSCC with LNM (Li L. et al., 2019). CKLF-like MARVEL transmembrane domain-containing 6 (CMTM6) was highly expressed in OSCC specimens. Besides, it was revealed that exosomal CMTM6 can be transferred to macrophages and regulate macrophage polarization (Pang et al., 2021). HSP90ɑ and HSP90β, as molecular chaperones, were markedly increased in EVs from metastatic cell lines and predicted poor prognosis of OSCC (Ono et al., 2018). Matrix metallopeptidases (MMPs) facilitate OSCC metastasis by degrading ECMs (Khan et al., 2020). MMP9 was strongly upregulated in exosomes from tumor cells (Qadir et al., 2018). Mechanistically, these results imply that proteins in EVs can serve as useful biomarkers of OSCC progression and metastasis. Apart from cargo proteins in EVs, some surface markers can also help with diagnosis. Despite CD63, CD81, and CD9 are downregulated in patients with oral cancer (OC) (Zlotogorski-Hurvitz et al., 2016). It was unveiled that life expectancy after surgery was correlated with the plasma level of exosomal CD63 (Rodríguez Zorrilla et al., 2019). Centrosomal protein (CEP55) was found on tumor-derived exosomes in HNCC, but not in primary human oral keratinocytes (Qadir et al., 2018). However, the specific mechanisms by which CD molecules and other transmembrane proteins are involved in OSCC progression remain unclear and further studies are warranted.

Taken together, these biomarkers have great potential for the diagnosis and prognosis of OSCC. Although our information about exosomal proteins, especially about exosomal surface proteins, is limited. CD markers lack stability and enough specificity. Future studies on exosomal proteins are needed to improve early diagnosis and prognosis in OSCC.

## 3 The role of EVs in the development of OSCC

**FIGURE 2:**
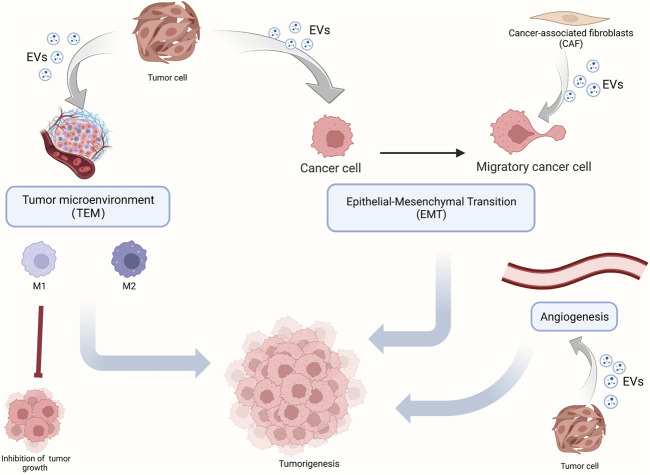
The role of EVs in OSCC tumorigenesis. OSCC tumorigenesis mainly includes the polarization of macrophages in the tumor microenvironment, epithelium-mesenchymal transformation, and angiogenesis. EVs carry molecular signals which play a regulating role.

Since the constituents of EVs can be used as diagnostic markers in OSCC, EVs may be involved in the development of OSCC. Herein, we review key components of OSCC development (Figure 2), such as TAM, polarization, angiogenesis, and EMT, and enumerate the role of EVs in these processes. In addition, we also elucidate the mechanism by which EVs promote chemotherapy resistance in OSCC and more comprehensively dissect the role of EVs in OSCC development.

### 3.1 The role of EVs to regulate TAM subtypes in OSCC

TME supports the growth of tumor cells. TME is composed of immune cells, stromal cells, extracellular matrix, and other components. The interaction between tumor cells and TME is crucial for tumor growth (Joyce and Pollard, 2009). TAMs are the main cells in TME and contribute to tumor development to varying degrees (Ruffell et al., 2012; Coussens et al., 2013; Mantovani et al., 2017). Recent studies have indicated that TAMs include M1 (classically activated macrophage) and M2 (alternatively activated macrophage) subtypes (Laviron and Boissonnas, 2019). OSCC cells and macrophages can release EVs that regulate TME. M1 macrophages have been described as the pro-inflammatory phenotype and play an important role in anti-microbial defense. M2 macrophages have been described as the anti-inflammatory phenotype and facilitate post-inflammatory tissue repair (Baig et al., 2020).

#### 3.1.1 EVs regulateM1 macrophage activation

Cancer cells release various EVs (Akers et al., 2013; Doyle and Wang, 2019). Several products of cancer cells can regulate macrophage polarization through the paracrine mechanism. EVs released by cancer cells can be internalized by macrophages and modulate the phenotypes of macrophages (Bellmunt et al., 2019; Baig et al., 2020). Many studies have suggested that M1 macrophages are antitumor phenotypes (Ai et al., 2021). M1 exosomal lncRNA HOTTIP inhibits HNSCC progression by competitively absorbing miRNA-19a-3p and miRNA-19b-3p and activating TLR5/NF-κB signaling pathway (Jiang et al., 2022). miRNA-9 is a key miRNA for tumor growth, metastasis, immunity, and radiosensitivity. Exosomes secreted by HPV + HNSCC contain miRNA-9, which downregulates PPARδ in macrophages, thereby inducing M1 polarization and increasing radiosensitivity in HNSCC (Tong et al., 2020). Recent studies have shown that M1-like cells also promote tumor development. The high invasiveness of M1-like TAM has been associated with the aggressive characteristics of some cancers. Similarly, M1-like TAM promoted tumor cell migration in OSCC (Gao et al., 2018; He Y. et al., 2020; Oshi et al., 2020). OSCC cells secrete EVs containing THBS1 that promotes macrophage polarization into M1-like cells. M1-like macrophages secrete IL-6, which activates Jak/STAT3 pathway in OSCC cells and promotes EMT, OSCC stemness, and THBS1 transcription (Xiao et al., 2018; You et al., 2022). In conclusion, the positive feedback loop between M1-like TAM and OSCC cells regulates EMT and cancer stemness (You et al., 2022). THBS1 is the most abundant protein secreted by OSCC. It is a multifunctional protein and an effective regulator of macrophage activation (Pal et al., 2016). In summary, M1-like macrophages are no longer considered the tumor-resistant phenotype, and they can promote tumor progression in certain circumstances.

#### 3.1.2 The role of EVs in M2-type macrophages activation

EVs provide a new method of information exchange between cells. Tumor cells interact with other cells in the TME by miRNA-riched exosomes (Brinton et al., 2015). TEM is mainly composed of macrophages, whose function can be regulated by OSCC-derived exosomes. M2-like macrophages promote tumor development (Baig et al., 2020). Endoplasmic reticulum stress was positively correlated with poor survival in OSCC patients. Many studies showed that programmed cell death-ligand 1 (PD-L1) prevents T cell activation and contributes to tumor immune escape (Xie F. et al., 2019; Daassi et al., 2020). Studies have shown that endoplasmic reticulum stress enhances PD-L1-rich exosome secretion by OSCC cells, thereby promoting M2 polarization of macrophages. M2-like macrophages impair the cytotoxic response of CD8^+^ T cell immune response and promote tumorigenesis (Yuan et al., 2022). CMTM6 is a key regulator of immune response in cancer. OSCC cells can release CMTM6-rich exosomes to activate ERK1/2 signaling pathway in macrophages, induce an M2-like phenotype, and promote tumor progression (Pang et al., 2021). The study also proved that CMTM6 deletion can reduce the proliferation and migration of OSCC cells, providing a new idea for treating OSCC.

### 3.2 Cargos in EVs to regulate angiogenesis of OSCC

EVs contain proteins, DNA, and miRNA. They precisely regulate tumor cell communication with neighboring cells and distant cells. Tumor cells can use EVs to reprogram signaling pathways in target cells and promote angiogenesis (Qadir et al., 2018). EVs can carry tumor antigens and specific proteins involved in vesicle formation and transportation (Baietti et al., 2012). Angiogenesis is essential for tumor development. Neovascularization provides oxygen supply and nutrients for the tumor cells. Increased angiogenesis and upregulation of VEGF reduce the overall survival rate of patients with OSCC (Kyzas et al., 2005). Therefore, understanding the mechanism of angiogenesis helps the targeted therapy in OSCC. Among 45 OSCC patients, higher levels of circulating microparticles (MPs) were significantly associated with tumor size, lymph node metastasis classification, vascular density, and VEGF expression. Circulating MPs isolated from OSCC patients can be internalized by human umbilical vein endothelial cells (HUVEC) and promote endothelial cell proliferation, migration, invasion, angiogenesis, and expression of pro-angiogenic factors (Ren et al., 2016). Studies have shown that EVs isolated from HNSCC cells can induce angiogenesis. EPHB2 carried by EVs can promote angiogenesis by inducing ephrin reverse signal transduction, thereby promoting tumor cell survival and metastasis (Sato et al., 2019). Many studies have shown that miRNAs carried by EVs can regulate angiogenesis in HNSCC (Panvongsa et al., 2022). AS a tumor suppressor gene, PTEN plays an important role in tumorigenesis. Exosomes carrying miRNA-130B-3P can promote angiogenesis by downregulating PTEN expression (Yan et al., 2021). OSCC-derived exosomes carry miRNA-221, which downregulates PIK3R1 and promotes HUVEC migration and duct formation (He S. et al., 2021). Many studies attempted to inhibit angiogenesis, thereby preventing OSCC development (Rosenberger et al., 2019). It has been suggested that exosomes of human deciduous stem cells carry miRNA100-5P and miRNA-1246, which inhibit angiogenesis (Liu P. et al., 2022).

### 3.3 The involvement of EVs in EMT of OSCC

EMT is a critical process in tumorigenesis. EMT is characterized by reduced expression of cell adhesion molecules and transformation of the cytoskeleton from keratin to vimentin. Epithelial cells are closely connected; however, mesenchymal cells have different morphologies, providing a higher invasion and migration capacity (Yeung and Yang, 2017). EMT is divided into three types. Type three EMT has been a research hotspot in recent years, as it is related to tumorigenesis. Identification and inhibition of key molecules in EMT is an important prerequisite for controlling tumor development. Tumor-derived exosomes (TDEs) can promote EMT, thereby enhancing invasion and migration capacity (Syn et al., 2016). CAFs also play a critical role in EMT. Many studies have shown that CAFs can interact with tumor cells via exosomes, and exosomal miRNAs can participate in oncogenesis (Yang et al., 2017). Transcription factors can directly modulate gene expression in EMT. Changes in RNA expression can also regulate EMT. The initiation and progression of EMT need different signaling pathways, and these pathways interact with each other as a network (Lamouille et al., 2014). As a bidirectional regulatory process, the interaction between EVs and tumor cells markedly differs based on the constituents of EVs. For instance, fibroblasts transfer exosomal miRNA-34a-5p to OSCC cells and may be involved in OSCC progression via the AKT/GSK-3β/β-catenin/Snail signaling pathway. These findings can help the treatment of OSCC. miRNA-34a-5p/AXL axis inhibitors may treat OSCC (Li et al., 2018). Currently, many studies are investigating EMT-based interventions to inhibit tumorigenesis.

### 3.4 EVs participates in the chemoresistance of OSCC

Chemoresistance can markedly undermine the efficacy of cisplatin and 5-FU, as the main chemotherapeutic agents for OSCC (Longley et al., 2003).

EVs can impair chemosensitivity and induce chemoresistance in OSCC through various mechanisms, thereby lowering the survival rate among OSCC patients. The constituents of exosomes, drug efflux by EVs, changes in vesicular pH, the anti-apoptotic signal transmitted by EVs, regulation of DNA repair mechanisms, immune response, and induction of cancer stemness and EMT by EVs are all involved in such outcomes (Law et al., 2021).

Many studies have shown that miRNAs mediate chemoresistance in tumor cells (Chen et al., 2014a; Chen et al., 2014b). OSCC cells with 5-FU resistance secreted APCDD1L-AS1-rich exosomes, which targeted miRNA-1224-5p and regulated miR-1224-5p/nuclear receptor binding SET domain protein 2 (NSD2) to induce 5-FU resistance (Li et al., 2021c). EMT enhances chemoresistance (Wang et al., 2016). Cells with acquired chemoresistance (cisRes90-OSCC) release exosomal miRNA-155, which targets FOXO3a and regulates EMT in OSCC cells (Kirave et al., 2020). Studies have shown that cisplatin-resistant cell lines (HSC-3-R, SCC-9-R) can transfer miRNA-21a to OSCC cells through exosomes. miRNA-21a induces cisplatin resistance by targeting PTEN and programmed cell death 4 (PDCD4) (Liu et al., 2017). Stromal cells such as macrophages can modulate the sensitivity of tumor cells to chemotherapeutic agents. It has been shown that macrophage-derived exosomes can reduce the sensitivity of OSCC cells to chemotherapeutic agents by activating the AKT/GSK-3β signaling pathway (Tomita et al., 2020).

## 4 EVs in multiple forms provide new ideas for OSCC treatment

The combination of surgery and chemotherapy is the most common and efficient therapeutic approach for patients with OSCC. In addition to chemotherapy resistance, which is common in OSCC, chemotherapeutics also cause major side effects, such as bone marrow depression, nephrotoxicity, gastrointestinal discomfort, skin and tongue lesions, and severe weight loss (Wang et al., 2015; Huang et al., 2020). Therefore, alternative therapeutic approaches are needed.

**FIGURE 3:**
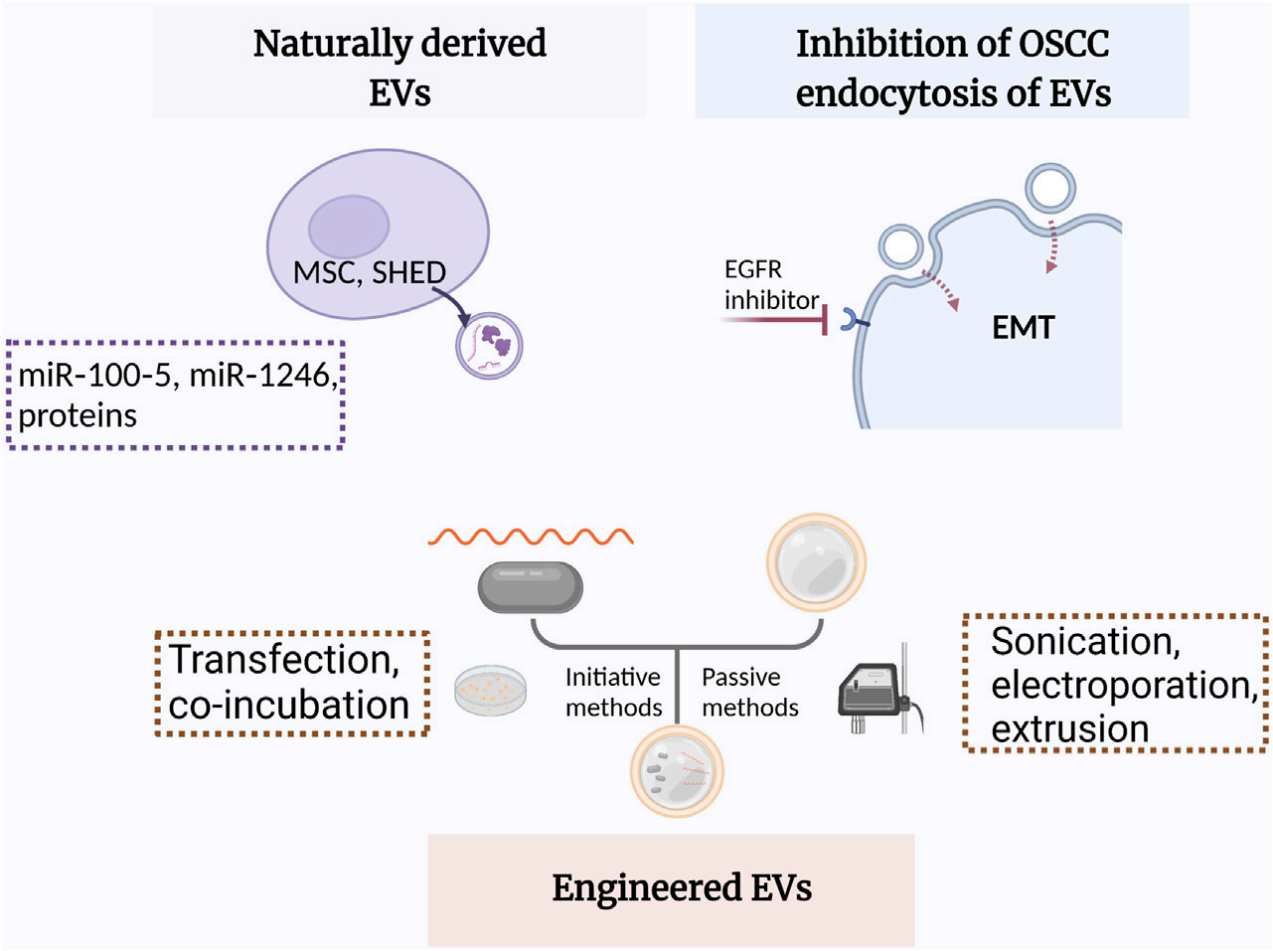
Schematic illustration of EVs applied in OSCC treatment. MSC, mesenchymal stem cells; SHEDs, stem cells of human exfoliated deciduous teeth; EGFR, epidermal growth factor receptor; EMT, epithelial-mesenchymal transition.

EVs from other cells may modulate some pathophysiological processes such as macrophage polarization, angiogenesis, and EMT through paracrine mechanism (Ohno et al., 2013; Kamerkar et al., 2017). Recently, inhibiting the ability of OSCC cells to endocytose EVs from TME has demonstrated satisfactory therapeutic efficacy (Fujiwara et al., 2018; Sasabe et al., 2022) (Figure 3).

### 4.1 Naturally derived EVs function as a double-edged sword

Mesenchymal stem cells (MSCs) can either suppress or promote immune response (Li et al., 2012; Liang et al., 2021). MSC-derived EVs can be used in cancer treatment. miR-16 from MSC-derived EVs can inhibit breast cancer growth by downregulating VEGF and subsequent angiogenesis (Lee et al., 2013). Similarly, exosomes derived from human exfoliated deciduous teeth (SHEDs) can attenuated angiogenesis in OSCC. A xenograft transplantation model indicated that miR-100-5p and miR-1246 carried by SHED-Exos can inhibited angiogenesis (Liu P. et al., 2022). However, MSC derived EVs are regarded as a double-edged sword due to promoting tumor progression. More studies are needed to overcome this issue.

### 4.2 Engineered EVs as a novel platform supplementary for targeted treatment in OSCC

Nanotechnology, particularly the development of different types of nanoparticles such as liposomes and biomimetic nanoparticles, has considerably improved the targeted treatment of tumors (Zhang et al., 2020). After being modified through initiative (transfection and co-incubation) or passive methods (freeze–thaw cycles, sonication, electroporation, and extrusion), nanoparticles can be loaded with chemotherapeutic agents, specific miRNAs, or other cargoes (Ebnoether and Muller, 2020; Zhang et al., 2021).

Modified EVs applied in other diseases can be similarly used for treating OSCC. Engineered EVs are now extensively used as drug carriers. We have previously constructed engineered neutrophil-derived apoptotic bodies (eNABs) loaded with hexyl 5-aminolevulinate hydrochloride (HAL) to ameliorate cardiac infarction (Bao et al., 2022). We also produced chimeric apoptotic bodies (cABs) preloaded with microRNA-21 to modulate inflammatio (Dou et al., 2020). As cancer immunotherapeutic drug carriers, dendritic cell-derived exosomes (DCs-Exo) loaded with antigens have been used for treating malignant melanoma (Nikfarjam et al., 2020). For specifically targeting cancer cells and preventing rapid clearance of drugs, specialized exosomes were constructed using iEXO-OXA platform and MSC-derived exosomes with superficial alteration with oxaliplatin (OXA) (Zhou et al., 2021) Through surface modification, CD44 facilitates lipid-mimetic-chains-grafted HA-modified EVs penetration into tumoral tissues and DOX delivery to tumor cells (Liu et al., 2019 ). Future improvements in EV design can improve the targeted therapy of OSCC. Different sources of biomimetic EVs have been used in previous studies to improve the efficiency of engineered EVs. Intriguingly, engineered bovine milk exosomes linked to doxorubicin via a special bond were packed with endoperoxides and chlorin e6 (Ce6) through a series of reactions. Producing exosomes by this method can overcome the low efficiency and ethical issues caused by using natural vesicles (Zhang et al., 2020).

In addition to promoting the selective uptake of therapeutic EVs by tumor cells, it is feasible to inhibit EVs endocytosis by phagocytic cells. Both upregulation of CD47 ( Mathieu et al., 2019 ) and downregulation of integrins α6β4 and αvβ5 on the surface of exosomes can prevent tumor cell metastasis (Hoshino et al., 2015).

Although much experience has been provided about engineered EVs in other cancers, few studies have been conducted in OSCC. Some researchers have found that the constituents of EVs can inhibit OSCC growth. Exosomal miR-34a-5p targets AXL, thereby inhibiting the proliferation and motility of OSCC cells through AKT/GSK-3β/β-catenin/Snail signaling pathway. But miR-34a-5p has low expression in OSCC cells. It suggests that EVs can be loaded with inhibitors of the miR-34a-5p/AXL axis to treat OSCC (Li, 2018). In fact, some miRs are enriched in exosomes to function efficiently. For instance, γδT cells infected with lenti-miR138 virus can produce miR-138-rich γδTDEs, which can be used in the treatment of OSCC. miR-138 targets PD-1 and CTLA-4 in CD8^+^ T cells and promotes anti-tumor immunity (L et al., 2019). Apart from miRs, some onco-miR inhibitors can be transported by EVs. Exosomes transfected with calcium chloride, a miR-155 suppressor, can upregulate FOXO3a and induce mesenchymal-epithelial transformation (MET) in cisplatin-resistant OSCC spheroids and *in vivo*, thereby reversing chemoresistance (Sayyed et al., 2021).

As mentioned previously, exosomes play a crucial role in the development of OSCC. Identifying the mechanism of exosome uptake can be promising in the treatment of OSCC. Epithelial growth factor receptor (EGFR) inhibition and knockdown both can inhibit exosome uptake by OSCC cells, thereby impeding OSCC invasion and progression. Similarly, OSCC development is influenced by the inhibitor of micropinocytosis (EIPA) (Sasabe et al., 2022). For example, cetuximab is assumed to be a therapeutic antibody for OSCC because it blocks EMT in EGFR-rich OSCC-EVs (Fujiwara et al., 2018).

Although most researchers believe that EVs are biocompatible even when administered repeatedly, their effects on other cells are still unclear. There are still many challenges, such as purification issues and immune properties, to be addressed before the clinical application of engineered EVs (Ketabat et al., 2019). Still, more progress in the field of engineered EVs is needed before their clinical application in OSCC.

## 5 The challenges for EVs in OSCC

### 5.1 The challenges for EVs in experimental research of OSCC

Currently, EVs can be classified by their origins, including cell culture-derived EVs, body fluid-derived EVs, and tissue-derived EVs (Ti-EVs). Cells lose their heterogeneity after several *in-vitro* passages, which can mask their original biological characteristics. In particular, a two-dimensional (2D) cultural environment can barely stimulate the *in-vivo* microenvironment (Jensen and Teng, 2020). Furthermore, EVs extracted at a specific time obscured the temporal nature of their actions, which could not reflect the course of the disease. In addition, EVs cannot be easily purified from the body fluid, and they are commonly contaminated with various proteins, such as serum proteins. However, Ti-EVs contain less impurities than body fluid-derived EVs. Studies have shown that Ti-EVs promote the development and metastasis of tumors (Costa-Silva et al., 2015; Hoshino et al., 2015; Liu and Cao, 2016). As mentioned previously, TEM-derived EVs are involved in the development of OSCC by regulating macrophage polarization, angiogenesis, and EMT (Xie et al., 2019b). Therefore, more attention should be paid to Ti-EVs in the future stage. There are still some challenges to be solved in the extraction of Ti-EVs, such as its challenges regarding their isolation and purification. It is necessary to develop standardized extraction methods, characterization methods, and titer evaluation methods to improve the reliability of Ti-EV (Li et al., 2021).

### 5.2 The challenges in the use of EVs for diagnosing OSCC

As previously mentioned, EVs can carry substances that contribute to the development and resistance of OSCC, making them novel diagnostic tools for OSCC (Han et al., 2018). Due to their presence in various body fluids, EVs are easy to acquire. Particularly, salivary EVs can be obtained at different stages of OSCC, without causing any discomfort to patients. This accelerates the use of EVs for diagnosing OSCC, following its progression, and developing more effective treatment methods.

However, there are still many problems to be solved before using EV as a new diagnostic method. The first problem is the lack of uniform diagnostic criteria. The contents of EVs derived from various cells are different. For instance, EVs derived from tumor parenchyma and those derived from immune cells in TEM are different (Liu P. et al., 2022). Secondly, most articles focus on the role of a single molecule. However, EVs have a variety of markers on their surface and carry various signaling molecules. Whether a single molecule or a combination of multiple molecules can be used as a diagnostic method needs to be clarified in the future. This also suggests that EVs can simultaneously target several signaling pathways.

Recently, some studies have shown that nano-flow cytometer (nFCM) can be used to detect DNA in EVs, which can be a more comprehensive method to determine the source of EVs for tumor diagnosis (Liu H. et al., 2022). In addition, it has been reported that microfluidic devices can be used to analyze and characterize single EVs to determine their exact physiological effects. It can become a new method of minimally invasive diagnosis of diseases (Bordanaba-Florit et al., 2021).

### 5.3 The challenges in the use of EVs for treating OSCC

EVs are increasingly recognized as a promising drug delivery system due to their unique physiological characteristics, including excellent biocompatibility, biodegradability, non-immunogenicity, and targeted delivery (Ha et al., 2016). However, EVs from various sources have their advantages and limitations. For instance, immune cell-derived EVs are highly targeted but not lethal; MSC-derived EVs have only anti-inflammatory and regenerative properties, and macrophage-derived EVs can regulate TEM and stimulate T cell activation. Therefore, engineering EVs, which can enhance their advantages and improve their limitations, has received much attention. Engineered EVs can be modified in an active or passive manner to enhance their therapeutic effects (Zhang et al., 2021). For example, car-T through the chimeric antigen receptor, improves the targeting specificity of T cells. Therefore, EVs can from car-T cells may become a new method of tumor treatment.

There are several challenges associated with EV-based treatment, including: 1) time-consuming and low-yield extraction process through ultra-centrifugation. 2) low efficiency of drug loading. 3) short duration of action *in vivo*. These issues necessitate further research and development, with a focus on constructing more effective engineered EVs.

## 6 Conclusion

EVs have emerged as a significant players in the diagnosis, development, drug resistance, and treatment of OSCC, and the precise mechanisms remain incompletely understood. This article aimed to review the involvement of EVs in the diagnosis, prognosis, tumorigenesis, drug resistance, and treatment of OSCC and outline the challenges associated with the use of EVs.

Recent studies indicated that EVs can be used as new diagnostic tools. As EVs can be easily obtained from saliva, blood, and other body fluids, the identification of their content is the least invasive diagnostic method. EVs are involved in many processes during OSCC development. They can regulate TAM polarization and promote or inhibit tumor development. EVs can also interact with tumor cells to promote angiogenesis, which is essential during tumorigenesis. Furthermore, EVs promote EMT, thereby facilitating tumor progression and invasion. EVs are also involved in drug resistance, leading to poor prognosis in OSCC. This article discussed natural EVs and engineered EVs. It also explained how to inhibit OSCC development by inhibiting EV endocytosis by tumor cells. Engineered vesicles can improve the treatment of OSCC.

In summary, EVs play a crucial role in the pathogenesis and progression of OSCC. Standardizing the diagnostic pattern and producing engineered vesicles for treating OSCC require more attention in the future. By addressing these challenges, we can accelerate the clinical translation of this promising biotechnology.
